# Budget Impact Analysis of Darbepoetin Alfa Every 3 Weeks versus Epoetin Alfa Every Week for Cancer Patients with Anemia due to the Effect of Concomitant Myelosuppressive Chemotherapy

**DOI:** 10.36469/9836

**Published:** 2015-11-16

**Authors:** November McGarvey, Hairong Xu

**Affiliations:** 1 Amgen Inc., Thousand Oaks, CA, USA

**Keywords:** chemotherapy-induced anemia, erythropoiesis-stimulating agent, budget impact

## Abstract

**Background:** Anemia is a common complication among patients with cancer receiving chemotherapy and can cause significant costs to health plans.

**Objective:** The objective of this study is to estimate the annual budget impact of drug treatment associated with treating cancer patients with anemia due to the effect of concomitant myelosuppressive chemotherapy (i.e., chemotherapy-induced anemia [CIA]) with erythropoiesis stimulating agents (ESAs), either darbepoetin alfa (DA) once every 3 weeks (Q3W) or epoetin alfa (EA) once every week (QW), for a large US health plan in 2014.

**Methods:** Using a patient database from a large US health plan in 2010 (n = 14 811 119), the potential CIA patient population was determined (1842 patients each per DA and EA). A budget impact of ESA treatment on this patient population in 2014 was calculated. The analysis assumed a minimum of 2 additional months of chemotherapy from initiation of the analysis. The 2014 Centers for Medicare and Medicaid Services (CMS) reimbursement rates used were: average sales price +12% of $3.68/mcg (DA) and $11.38/1000 IU (EA), and office-based injection cost of $25.08.

**Results:** The estimated 2014 annual average drug costs per patient with CIA were $5520 (DA) and $5833 (EA). Annual average drug costs for administrations were estimated at $100 (DA) and $301 (EA) for 2014. Per member per year (PMPY) costs for patients with CIA were estimated at $5620 (DA) and $6134 (EA) for 2014. The annual total costs per CIA population (n=1842) were estimated at $10 352 629 (DA) and $11 298 798 (EA) for 2014.

**Conclusion:** DA Q3W has the potential to provide cost savings over EA QW in terms of annual average drug cost per patient with CIA ($313 savings), PMPY costs for patients with CIA ($514 savings), and total cost per CIA population ($946 169 savings).

### BACKGROUND

Anemia is characterized by a decrease in the number of red blood cells (RBCs) or hemoglobin (Hb), causing a decreased ability for the blood to carry oxygen to body tissues. It often develops in patients with cancer due to the disease itself or because common cancer treatments such as chemotherapy suppress the bone marrow. The World Health Organization (WHO) defines anemia as a Hb level of <13.0 g/dL in men and <12.0 g/dL in women[1] and the National Cancer Institute (NCI) defines it as a Hb level of <14.0 g/dL in men and <12.0 in women.[2] Anemia prevalence is 30%-90% in cancer patients[3] and the incidence of anemia may be as high as 70%-90% in patients receiving myelosuppresive chemotherapy and/or radiation therapy.[4]

Anemia may negatively impact cancer patients, their caregivers, and health plans. Clinical symptoms for individuals with anemia can include the following: fatigue, tachycardia, cognitive impairments, shortness of breath, depression, dizziness, chest pain, weakness, loss of appetite, and headache.[5] Some common anemia symptoms (e.g., fatigue and dyspnea on exertion) may interfere with an individual's ability to perform normal activities such as self-care, work, and care of one's family[6] and decrease health-related quality of life (HRQoL).[5-13] Patients with anemia due to the effect of concomitant myelosuppressive chemotherapy (CIA) may need assistance from informal caregivers to perform normal daily activities and activities related to their treatment (e.g., travel assistance to clinic visits).[14-20] In addition to patient and caregiver burden, anemia can cause significant costs to health plans.[21-23] The average costs for patients with anemia may be as much as twice that of those of their non-anemic counterparts.[23]

Therapies for treating CIA include RBC transfusions and erythropoiesis stimulating agents (ESAs) such as darbepoetin alfa (DA) or epoetin alfa (EA). Treatment with RBC transfusions is generally considered a "rescue" therapy only for patients with severe anemia.[24, 25] RBC transfusions are costly, only have a transient effect, and do not address the causes of anemia. ESAs treat anemia by increasing RBC production in the bone marrow using the same molecular mechanism as endogenous erythropoietin. The ESAs, DA and EA, have similar safety and efficacy profiles.[26-30] Economic comparisons of the ESA regimens may help assist in formulary decision making. The common administration of the ESAs differ with DA potentially being administered once every 3 weeks (Q3W) and EA once every week (QW). A Q3W administration schedule may be advantageous as this schedule may allow for better synchronization with ongoing cancer treatments. A budget impact model was created in order to help evaluate the impact of these different administration schedules of EA and DA to a US health plan. The objective of the model was to estimate the annual budget impact in 2014 of drug treatment associated with treating CIA with ESAs, either DA Q3W or EA QW, for a large US health plan.

### METHODS

A retrospective analysis of administrative claims data for a large US commercial health plan was used as the basis for population inputs into the estimation of the budget impact of ESA treatment on patients with CIA in 2014. The administrative claims data included 14 811 119 US commercially-insured individuals from January 1, 2010 through December 31, 2010. Eligible claims were limited to those of patients between the ages of 18 and 64, who had a physician diagnosis of non-myeloid malignancies, received chemotherapy during the intake period, and had at least 12 months of medical and pharmacy benefit eligibility prior to the cancer diagnosis. The retrospective claims analysis provided the following input estimates: prevalence of metastatic, nonmyeloid malignancies = 1.48%; patients with metastatic, nonmyeloid malignancies receiving cytotoxic chemotherapy = 23.40%; cytotoxic chemotherapy patients with anemia in this analysis = 3.70%; cytotoxic chemotherapy patients currently receiving treatment = 97.00% (Calculations in Table 1). Using the rounded up product of these inputs with the total number of enrolees resulted in an estimate of 1842 CIA

patients receiving ESA treatment in the plan.

**Table 1.** Inputs to Calculations on Estimated Number of CIA Patients Receiving ESA Treatment

| Model Input | Calculation | Value |
|---|---|---|
| Total number enrollees | | 14 811 119 |
| Prevalence of metastatic, nonmyeloid malignancies | [Total patients with metastatic, nonmyeloid malignancies (219 070) / Total Enrollees (14 811 119)] x 100% | 1.48% |
| Percentage patients with metastatic, nonmyeloid malignancies receiving cytoxic chemotherapy | [Total patients receiving cytoxic chemotherapy (42 049) / Total patients with metastatic, nonmyeloid malignancies with at least 12 months continuous enrollment (179 822)] x 100% | 23.40% |
| Prevalence of anemia in cytoxic chemotherapy patients | [Total patients with ICD-9 Code for CIA (1554)/Total patients receiving cytoxic chemotherapy (42 049)] x 100% | 3.70% |
| Prevalence of cytoxic chemotherapy patients with anemia currently receiving treatment with DA or EA | [Total patients with ≥ 1 medical or pharmacy claim for SA(1513)/ Total patients with ICD-9 Code for CIA (1554)] x 100% | 97.00% |
| Estimated Number of CIA Patients Receiving ESA Treatment | Rounded up [Total number enrollees (14 811 119) x Prevalence of metastatic, nonmyeloid malignancies (0.0148) x Patients with metastatic, nonmyeloid, malignancies receiving cytotoxic chemotherapy (0.234) x Cytotoxic chemotherapy patients with anemia (.037) x Prevalence of cytoxic chemotherapy patients with anemia currently receiving treatment with DA or EA (0.97)] | 1842 |

CIA: chemotherapy-induced anemia; DA: darbepoetin alfa; EA: epoetin alfa; ESA: erythropoiesis stimulating agent

For the budget impact model, a population of 1842 patients each per DA and EA is utilized. Default dose values in the analysis are based on previous literature reporting of average dose. Average doses are used rather than published label doses to better reflect a real-world situation. The doses are as follows: DA dose of 375 mcg every three weeks for a course of four injections[31] and EA dose of 42 714 IU every week for a course of 12 injections.[32] In order to reflect the appropriate time horizon based on these average ESA doses, the budget impact analysis reflects a 12 week period. This budget impact analysis also assumes that upon ESA treatment initiation there is a minimum of two additional months of planned chemotherapy. The 2014 Centers for Medicare and Medicaid Services (CMS) reimbursement rates used for the analysis were the published average sales price (ASP) of +12% of $3.68 per mcg (DA) and $11.38 per 1,000 IU (EA) and an office-based injection cost of $25.08.[33] Budget impact model inputs are found in Table 2.

The total drug cost is calculated by multiplying the 2014 CMS ASP +12% amount with the dose with the total number of injections during treatment (Total drug cost = ASP +12% x dose x number of injections). The total drug administration cost is calculated by multiplying the 2014 CMS injection cost with the number of injections (Total drug administration cost = injection cost x number of injections). The per member per year (PMPY) cost is calculated as the total drug cost added to the total drug administration cost. The annual total costs per population are determined through multiplying the PMPY costs by the total population of patients with CIA receiving ESA treatment.

**Table 2.** Budget Impact Model Inputs

| | DA* Regimen | EA† Regimen |
|---|---|---|
| Dose | 375.0 mcg | 42 714 IU |
| Frequency of Administration | every 3 weeks | 1 time per week |
| Price | $3.68 / mcg | $11.38 / 1000 IU |
| Time Horizon | 12 weeks | 12 weeks |
| Number of Injections | 4 | 12 |
| Cost of Injection | $25.08 | $25.08 |
| % Population | 100% | 100% |
| Population (n) | 1842 | 1842 |

* DA = Darbepoetin alfa
† EA = Epoetin alfa

### RESULTS

Costs estimates for 2014 were calculated for the total drug cost, the total drug administration cost, the total PMPY cost, and the annual total cost per CIA population (Table 3). All costs calculated were higher for EA QW compared to DA Q3W. The annual average drug costs per patient with CIA were $5520 (DA Q3W) and $5833 (EA QW). Average drug costs for administrations among patients with CIA were $100 (DA Q3W) and $301 (EA QW). Per member per year (PMPY) costs for patients with CIA were $5620 (DA Q3W) and $6134 (EA QW). The annual total costs per CIA population (n=1842) were $10 352 629 (DA Q3W) and $11 298 798 (EA QW).

**Table 3.** Scenarios of Total Cost Based on DA* or EA† Dosing Regimen

| **DA Regimen** | | | | | | | | | | |
|---|---|---|---|---|---|---|---|---|---|---|
| Dose | | Cost DA | | # injections | | Cost Injection | | Total drug cost[a] | | Total drug administration cost[b] |
| 375.0 mcg | x | $3.68/mcg | x | 4 | x | $25.08 | = | $5520 | + | $100 |
| | | | | | | | | | | |
| | a | | | | b | | | | | |
| | | | | | | Total PMPY for patients with CIA cost | = | $5620 | | |
| | | | | | | | | | | |
| | | | | | | Annual total costs per CIA population (n=1842) | = | $10 352 629 | | |
| **EA Regimen** | | | | | | | | | | |
| Dose | | Cost EA | | # injections | | Cost Injection | | Total drug cost[a] | | Total drug administration cost[b] |
| 42 714 IU | x | $11.38/1000 IU | x | 12 | x | $25.08 | = | $5833 | + | $301 |
| | | | | | | | | | | |
| | a | | | | b | | | | | |
| | | | | | | Total PMPY for patients with CIA cost | = | $6134 | | |
| | | | | | | | | | | |
| | | | | | | Annual total costs per CIA population (n=1842) | = | $11 298 798 | | |

Note: Description of cost inputs and calculations are found in the methods section
* DA = Darbepoetin alfa
† EA = Epoetin alfa
# PMPY = Per member per year

## DISCUSSION

### Comparison to Previous Research

This budget impact analysis follows trends found by other previous economic analyses comparing DA and EA, with DA having some cost advantage over EA or being relatively comparable in price.[34-36] Similar to this more current budget impact model, Rubin and colleagues conducted a budget impact analysis to evaluate the economic impact of ESA therapy from a US health plan perspective in patients with non-myeloid malignancies and CIA.[35] The analysis focused on the estimation of direct medical costs that are relevant to a third party payer and compared the total direct medical costs of ESA treatments (using 2007 drug acquisition and administration costs and mean doses of 375.6 mcg DA Q3W and 43 187 IU EA QW). This analysis compared a DA Q3W dose and administration schedule with an EA QW dose and administration schedule over a 16-week time horizon. Based on an estimate of 2735 CIA patients in a health plan population of 2.17 million, the analysis found a per-patient budget impact of $8544 and $8667 for DA Q3W and EA QW, respectively. Per member per month costs were $0.90 for DA Q3W and $0.91 for EA QW.

Song and colleagues also conducted a cost comparison of DA and EA through a retrospective analysis of a claims database of patients continuously enrolled from December 2004 through June 2005 meeting inclusion criteria.[36] Episodes of care were constructed in 324 DA and 342 EA users with costs based on reimbursed costs in the claims database. This cost analysis included all the DA and EA administration schedules in the claims data selected: DA QW, once every two weeks (Q2W), as well as Q3W dosing administration). After accounting for duration of clinical benefit, Song and colleagues also found some cost savings for DA over EA with results that the average weekly cost of DA was significantly lower than that of EA ($619 versus $940; $p < 0.001$).

One additional older analysis conducted by Reed and colleagues examined costs between 40 000 IU EA QW versus 200 mcg DA Q2W among 358 patients with solid-tumor cancers and CIA over the course of 16-weeks in an open-label, multicenter, randomized trial.[34] In contrast to our cost analysis, the Rubin and colleagues' analysis, and the Song and colleagues' analysis, Reed and colleagues did not assess EA Q3W dosing administration and instead examined off-label Q2W dosing administration. Mean total costs calculations included costs for study medications and their administration, inpatient care, transfusions, unplanned radiation therapy, hematology and laboratory services, chemotherapy and non-chemotherapy drugs and indirect costs in 2004/2005 US dollar values. Overall, the mean total costs were relatively similar with DA Q2W slightly less costly that EA QW ($14 976 EA QW arm compared with $14 101 DA Q2W arm, a difference of $875 US dollars (95% CI for difference -849, 2607)). Ninety-eight percent of the cost difference was attributable to higher inpatient costs in the EA QW arm.

### Limitations

This budget impact analysis is subject to some limitations. As the basis of this model relies on US data inputs, the data generalizability is limited to US health plans similar to that proposed in the model. Additionally, the model is dependent on the relative accuracy of estimates of inputs into the model. Efforts were made to minimize potential issues of non-representative model inputs through use of estimates based on large US health plan with the CIA population of interest, real-world doses, and published costs for the time period of interest. The real world doses differ from the prescribing information on the DA and EA labels (DA Q3W on label 500 mcg versus real world dose of 375 mcg and EA QW on label 40 000 IU versus real world dose of 42 714 IU). While the use of real-world doses in this analysis may favor DA Q3W in terms of the drug costs, savings in costs related to reduced number of administrations for DA Q3W are not impacted. As the objective of this analysis is to examine costs from a payer's perspective, use of real world doses to demonstrate estimated actual costs are more relevant. The model outcomes are limited to select cost outputs related to health plan payer interests. The model does not include medical costs related to the management of treatment-related or nontreatment-related adverse events, Hb monitoring, laboratory tests, blood transfusions, hospitalizations, potential improvements in long-term clinical outcomes, or indirect costs (such as lost productivity). However, most of these additional cost components are likely to equally impact both DA Q3W and EA QW and are not expected to greatly affect the overall comparison of the products in the budget impact analysis. Also, this budget impact analysis does not measure efficacy endpoints (such as an increase or decrease in Hb level or in RBC transfusion requirements) or the economic impact of the endpoints for both products.

#### Future Research Considerations

As noted in the limitations, this economic analysis like others, has not fully accounted for factors related to indirect costs (e.g., costs associated with travel to treatment, patient satisfaction with treatment, patient quality of life), caregiver costs (e.g., caregiver quality of life, health, and time), employer costs (e.g., work productivity), and societal costs (e.g., national healthcare burden and costs). More comprehensive budget impact analyses accounting for these types of factors are needed to better inform differentiation of DA and EA regimens based on a wider scope of what ESA treatment costs mean.

Further exploration is also needed on the potential cost benefits related to the extended administration schedule of DA Q3W. The extended administration schedule of DA Q3W may be advantageous as it allows for synchronization of patients' anemia treatment with ongoing cancer treatments. Synchronization of treatments may reduce the number of visits and blood tests and allow for less time spent by the patient and clinician for drug administration and treatment. Reitan and colleagues conducted a retrospective chart review of data from 2007 through 2010 to collect information on the number and types of visits in two private oncology practices for patients receiving a complete course of myelosuppressive chemotherapy. The chart review demonstrated that the mean total staff time expended per chemotherapy course was greater for patients receiving DA QW versus DA Q3W. Patients receiving QW dosing had greater projected mean labor costs than those receiving Q3W ($38.16 vs. $31.20). Future ESA budget impact analyses may benefit from inclusion of administration and labor cost saving associated with synchronization of treatment.

### CONCLUSIONS

DA Q3W has the potential to provide cost savings over EA QW in terms of annual average drug cost per patient with CIA ($313 savings), cost per member per year for patients with CIA ($514 savings), and total cost per CIA population ($946 169 savings). This budget impact model may aid in providing cost comparisons important for formulary decision making. Future budget impact models may provide a more comprehensive view of costs through inclusion of a wider array of inputs (e.g., indirect costs, caregiver costs, employer costs, and societal costs).

#### Declaration of Funding

Amgen, Inc. provided the financial support to conduct the budget impact analysis reported on in this manuscript. The preparation of this manuscript involved an employee of Amgen, Inc. and a consultant assigned to Amgen, Inc.

### Declaration of Financial/Other Relationships

November McGarvey is a health economics consultant assigned to Amgen, Inc. and received research support from Amgen, Inc. Hairong Xu is a current employee and stockholder of Amgen, Inc.
